# Machine learning-based spatial data development for optimizing astronomical observatory sites in Indonesia

**DOI:** 10.1371/journal.pone.0293190

**Published:** 2023-10-20

**Authors:** Anjar Dimara Sakti, Muhammad Rizky Zakiar, Cokro Santoso, Nila Armelia Windasari, Anton Timur Jaelani, Seny Damayanti, Tania Septi Anggraini, Anissa Dicky Putri, Delik Hudalah, Albertus Deliar

**Affiliations:** 1 Remote Sensing and Geographic Information Science Research Group, Faculty of Earth Sciences and Technology, Institut Teknologi Bandung, Bandung, Indonesia; 2 Center for Remote Sensing, Institut Teknologi Bandung, Bandung, Indonesia; 3 Business Strategy and Marketing Research Group, School of Business and Management, Institut Teknologi Bandung, Bandung, Indonesia; 4 Astronomy Research Group and Bosscha Observatory, Faculty of Mathematics and Natural Sciences, Institut Teknologi Bandung, Bandung, Indonesia; 5 U-CoE AI-VLB, Institut Teknologi Bandung, Bandung, Indonesia; 6 Air and Waste Management Research Group, Faculty of Civil and Environmental Engineering, Institut Teknologi Bandung, Bandung, Indonesia; 7 Regional and Rural Planning Research Group, School of Architecture Planning and Policy Development, Institut Teknologi Bandung, Bandung, Indonesia; Arab Academy for Science Technology and Maritime Transport, EGYPT

## Abstract

Astronomical observatory construction plays an essential role in astronomy research, education, and tourism development worldwide. This study develops siting distribution scenarios for astronomical observatory locations in Indonesia using a suitability analysis by integrating the physical and atmospheric observatory suitability indexes, machine learning models, and long-term climate models. Subsequently, potential sites are equalized based on longitude and latitude zonal divisions considering air pollution disturbance risks. The study novelty comes from the integrated model development of physical and socio-economic factors, dynamic spatiotemporal analysis of atmospheric factors, and the consideration of equitable low air-pollution-disturbance-risk distribution in optimal country-level observatory construction scenarios. Generally, Indonesia comprises high suitability index and low multi-source air pollution risk areas, although some area has high astronomical suitability and high–medium air pollution risk. Most of Java, the east coast of Sumatra, and the west and south coasts of Kalimantan demonstrate "low astronomical suitability–high air pollution risk.” A total of eighteen locations are recommended for new observatories, of which five, one, three, four, two, and three are on Sumatra, Java, Kalimantan, Nusa Tenggara, Sulawesi, and Papua, respectively. This study provides a comprehensive approach to determine the optimal observatory construction site to optimize the potential of astronomical activities.

## Introduction

Since the development of astronomical observatories in 1560, astronomical observation activities have undergone extensive development worldwide [1]. The modernization and equitable distribution of observatory construction play an important role in developing research, education, and global tourism in astronomy [2–4]. One of the important aspects of optimizing astronomical observation activities is planning the best location for constructing the observatory [5]. Evaluation of the characteristics and environmental conditions of the observatory construction is required to optimize observations of astronomical phenomena [3]. These various factors, e.g., climatic conditions, atmospheric conditions, elevation, access to infrastructure, and human activities, need to be considered to optimize the function of the optical telescope [6, 7]. For example, one impact of improving infrastructure and human activities is the phenomenon of light pollution, which is a crucial factor hindering the observation of astronomical objects [8, 9].

Indonesia has great potential for the development of astronomical activities for observation of both the northern and the southern hemispheres due to its unique location at the equator. In addition, the long longitude, if it can be utilized with the construction of an optimally distributed observatory in the right location, will increase the total monitoring time of particular astronomical phenomena e.g. transient objects. This big potential can be used to develop an astronomical tourism industry that offers various visual experiences to the observations. However, Indonesia currently has only two main research observatories, the Bosscha Observatory and the ITERA Astronomical Observatory (IAO), and one under construction, the Timau National Observatory, located in Timau. The development of modern astronomy in Indonesia has progressed since the founding of the Bosscha Observatory in Lembang in 1923 [10]. However, since 1990, the sky quality at the Bosscha Observatory has declined due to development and human activities, causing light pollution [11, 12]. Therefore, studies to determine the optimal site selection for constructing new observatories in Indonesia need to be developed further to take advantage of this potential.

The suitability of the optical astronomical observatory site location can generally be determined by directly observing the observatory location by measuring the sky quality parameters using the differential image motion monitor (DIMM) method. Site testing using DIMM technology has been conducted in several previous studies, such as by Liu et al. [13] in Xinglong, China, and Nasiri et al. [14] in Iran. In addition to DIMMs, a photometer sky quality meter (SQM) can also be used to measure night sky brightness (NSB) to see the effect of light pollution e.g., in Bandung, Indonesia by Herdiwijaya [9]) and in Madrid by Zamorano et al. [15]. Both methods of direct measurement have the advantage of accurately determining the sky quality for probable site location. However, they need the best candidates first before conducting the direct measurement; therefore, they are not cost-efficient, and the coverage area is minimal. In addition to the direct method, there is also an indirect observation method using remote sensing technology that can determine the suitability of the location and supporting factors through spatial analysis. This method is cost-efficient and can cover a wide area. However, it also depends on the spatial and temporal resolution of the available spatial data. Previous studies have applied remote sensing approaches to determine the best location for observatories, such as the study by Koc-San et al. [16] in Antalya Turki, the study by Umar et al. [17] in the Malaysian Peninsula, and the study by Daniyal and Kazmi [18] in Pakistan using the Analytic Hierarchy Process (AHP) method to weight each parameter. Apart from AHP, the use of the multi-criteria decision analysis (MCDA) method was also tested in western Antarctica using regional climate models [19], and in studies using disaster risk parameters located in Alentejo, Portugal [20], Turkey [21], and Australia [22]. Similar research in Indonesia has also been carried out using spatial-temporal variations by Hidayat et al. [3]. Some global studies have analyzed observatories’ suitability using atmospheric and elevation parameters, as in Aksaker et al. [5]. In general, previous studies have not grouped the physical factors of the Earth’s condition, demographic–economic, and climatic conditions. A better integration process between the Earth’s physical factors, which tend to be static, with climate factors, which tend to be dynamic, is expected to increase the sensitivity related to the analysis of the optimal suitability level of the observatory location.

This study aims to develop a scenario for the distribution of optimal locations for the construction of infrastructure for astronomical research observatories in Indonesia. To achieve this goal, several supporting objectives include developing an observatory suitability index based on machine learning approaches of earth’s physical, demographic–economic, and atmospheric-environmental factors, identifying the location of observatory construction based on the division of longitude and latitude zones and considering the risk of air pollution disturbance. Physical parameters include elevation; demographic–economic factors; including population, human activities, road, and electricity accessibility; and light pollution. Atmospheric-environmental parameters included rainfall, cloud cover, wind speed, aerosol optical depth (AOD), and precipitable water vapor (PWV). The novelty of this study is related to the integration process of physical, socio-economic, and atmospheric factors based on machine learning remote sensing data and long-term climate models, as well as the consideration of equitable distribution with a low risk of air pollution disturbance in determining the optimal scenario for equitable distribution of observatories at the country level. Through this study, regulators and astronomers can globally apply a comprehensive approach to determine the best observatory construction location to optimize the research, education, and astro-tourism potential.

## Material and methods

**Fig 1:**
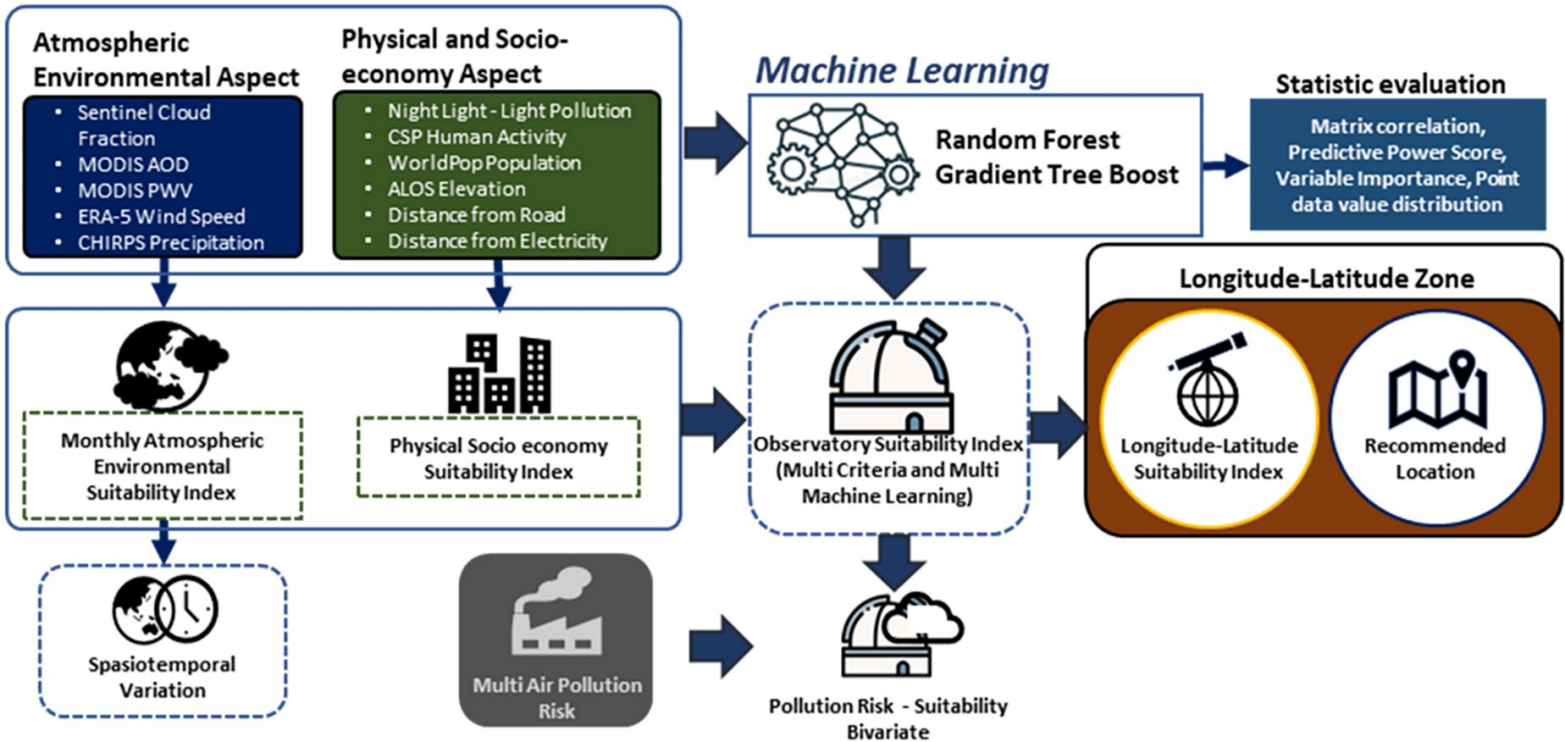
The scheme of the methodology used in this study.

In general, the methodology for data processing is divided into five main stages: the development of the observatory suitability index from physical parameters, the development of the atmospheric suitability index from the atmospheric parameters, the combined index data processing based on multi-criteria and multi-machine learning approaches, and the distribution of zoning divisions based on longitude and latitude. For discussion, a three-point observatory evaluation was performed on the spatiotemporal analysis of six dynamic atmospheric parameters including the integration process with multi-pollution risk data to obtain the optimal location for the distribution of observatory development recommendations. The general methodology is illustrated in Fig 1. The data have various spatial resolutions, so it is necessary to transform them into a single resolution before data integration, which was achieved using nearest neighbor resampling to maintain a uniform spatial resolution target of 1 km [5].

### Data used in this study

**Fig 2:**
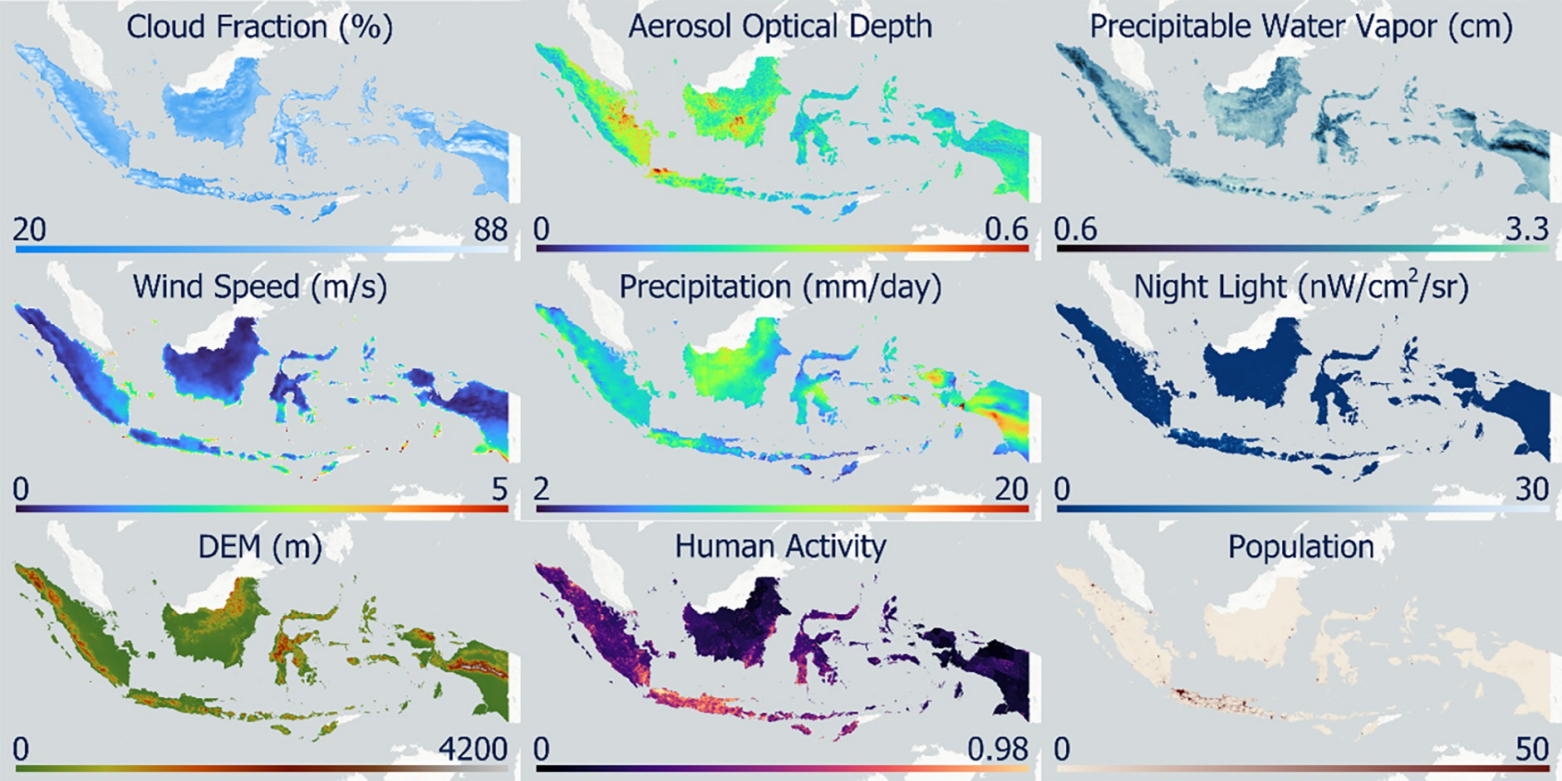
Raster data visualization used in this study.

The study area was Indonesia, which has seventeen domed observatories: three main research observatories (Bosscha, ITERA, and Timau) and about fourteen education observatories used for specific research purposes e.g., crescent moon observation. The data used in this study is presented in Table 1. The data grouping based on the objectives can be grouped into physical and atmospheric factors. Based on the dataset type, the data can be divided into vectors, static rasters, and dynamic rasters. The visualization of the nine raster datasets used in this study is shown in Fig 2.

**Table 1:** Summary of the data type and format.

Data	Product	Year data	Type-Resolution	Reference
Road Network	Global Road Inventory	2018	Vector	[23]
Electrical Network	Global Energy Infrastructure	2020	Vector	[24]
Human Activity	Global Human Modification	2016	Raster-1 km	[25]
Human Population	WorldPop	2020	Raster-100 m	[26]
Light Pollution	VIIRS Nighttime	2014–2021	Raster-463 m	[27]
Elevation	ALOS JAXA	2021	Raster-30 m	[28]
Cloud Fraction	Sentinel-5P ESA	2018–2021	Raster-1.11 km	[29]
Aerosol Optical Depth	MODIS MAIAC	2000–2021	Raster-1 km	[30]
Precipitable Water Vapor	MODIS MAIAC	2000–2021	Raster-1 km	
Wind Speed	ERA5	2000–2021	Raster-11.13 km	[31]
Precipitation	CHIRPS	2000–2021	Raster-5.56 km	[32]
Administration Data	GADM	2022	Vector	[33]
Air Pollution Risk	ITB	2019–2020	Raster-1 km	[34]

The vector data used in this study were the Indonesian administrative boundaries, road networks, and electrical networks. Indonesia’s territorial boundaries determine the administrative area of the Indonesian state. The data used were a product of the Database of Global Administrative Areas [33]. Road data throughout Indonesia were sourced from GRIP GLOBIO in 2018 in the form of vector data with line data type [23]. The results of the road model in Meijer et al. [23] had a coefficient of determination that reached 0.9 in the total value of the length of each country’s roads to population density, gross domestic product (GDP), and Organization for Economic Co-operation and Development (OECD) memberships. The second vector data were the electricity grid data sourced from the GEI Gridfinder 2020 [24], and the first were composite data in the form of vector data (lines) that provided a global power system with an open license. The power grid data had a model accuracy of 75% for all datasets validated in several countries. Road and electricity network data were used to determine the location of observatories with high accessibility to reduce observatory construction costs [18].

Static raster data, which are in the form of data on human activity and population, light pollution, and elevation, were not used temporally. The three datasets were integrated to determine the physical index for the observatory. Human activity data were in the form of an index ranging from 0 to 1 with a spatial resolution of 1 km, derived from global human modification [25]. The five main types of anthropogenic data considered in the study by Kennedy et al. [25] were data on settlements, agriculture, transportation, mining and energy production, and electricity infrastructure. Human population data for 2020 were obtained from WorldPop, with a spatial resolution of 100 m [26]. World population data come from population census data that are matched by administrative units into 100 m grid cells through a machine learning approach to determine the relationship between population density and various geospatial covariates. Human activity and population data were used because the optimal observatory location is one with a small population and minimal human activity [17, 35]. In addition, the light pollution data came from the night light radiance of the VIIRS image sourced from the Colorado School of Mines, with a spatial resolution of 463 m and monthly temporal resolution [27]. These data have units of nanoWatts/cm2/sr with a minimum value of -1.5 and a maximum value of 193564.92. Light pollution data were used because light pollution interferes with astronomical observations [6]. The last static raster data were elevation data from the ALOS DSM product sourced from the Japan Aerospace Exploration Agency with a spatial resolution of 30 m v3.2 [28]. Digital surface model (DSM) data were used because the thickness of the atmosphere decreases with increasing elevation, which affects the observatory suitability index [21].

In the dynamic raster data, temporal variation was used to determine the dynamics of change based on time. The data grouped under this type included cloud cover, AOD, PWV, rainfall, and wind speed. Five data points were used to determine the atmospheric index of the observatory from 2019 to 2021. Cloud cover data were obtained from the Sentinel-5P satellite from ESA in 2018–2021 with a spatial resolution of 1.11 km and daily temporal resolution [29]. The cloud cover data had a number fraction ranging from 0 to 1. The cloud cover factor is used to determine the location of the observatory because the presence of clouds interferes with astronomical observations [18]. Aerosol and water vapor data were sourced from MODIS MAIAC with 1 km spatial resolution and daily temporal resolution [30]. Aerosol and water vapor data must be considered because these parameters can interfere with sky transparency [16, 22]. Climate Hazards Group InfraRed Precipitation with Station (CHIRPS) data are global rainfall data for more than 30 years obtained by integrating satellite image data and in-situ station data to produce a time series of rainfall with units of mm/day and a spatial resolution of 5.56 km [32]. Locations with low rainfall intensities are highly suitable for observatory areas [17]. The wind speed data is derived from long-term climate modeling from the European Center for Medium-Range Weather Forecast (ECMWF) with a spatial resolution of 11.13 km and monthly temporal resolution [31]. The wind speed component used was the resultant wind speed, which was processed from the U and V components of the wind data. Wind speed data must be considered because high wind speeds affect atmospheric stability [5]. In addition to these five datasets, dynamic raster data uses air pollution risk data from Sakti et al. [34] in the form of air pollution risk data in Southeast Asia by considering the relationship between CO, NO_2_, and SO_2_ gases and socio-economic factors. These data were used to obtain priority areas for future observatory development that are optimal and minimize the risk of high air pollution.

### Observatory suitability index development

The observatory suitability index was developed by integrating the results of the physical index and atmospheric index products. To detect physical parameters, this study uses spatial analysis to determine the index based on the physical parameters of the observatory in the form of the Earth’s physical and socio-economic parameters. The general steps are calculating light pollution, proximity infrastructure analysis, and integrating physical factors. The first step is to develop a value for the nighttime light brightness to detect the level of light pollution. This product is processed from the nightlight radiance value of the VIIRS image, which is converted to the NSB value using Eq 1. The second stage is to perform a proximity analysis for road and electricity network data to obtain the distance value between the road and electricity. The third stage is the physical parameter integration, consisting of the distance from the road network and electricity obtained from the results of the first stage, light pollution calculated in the second stage, human activities, human population, and elevation. Scoring is then performed for each parameter, which is reclassified into five classes using quantile classification. According to Herdiwijaya [9], NSB can be reclassified into five classes. According to Kang et al. [36], elevation can be classified into five classes. The six parameters were summed to obtain the observatory physical index using Eq 2 with IFO, PO, MO, JA, LI, DEM, and NSB sequentially, which were the observatory physical index, population, human activity, distance from the road network, distance from the power grid, elevation, and light pollution.

$$NSB=\sum_{i=0}^{n}20-1.9log\;(VIIRS)$$ (1)

$$IFO=\sum_{i=0}^{n}{PO}_{i}+{MO}_{i}+{JA}_{i}+{LI}_{i}+{DEM}_{i}+{NSB}_{i}$$ (2)

Five dynamic parameters are used to develop the atmospheric observatory index: cloud cover, aerosol optical depth, water vapor, wind speed, and rainfall. The data used to calculate the atmospheric index are the averages for 2019–2021. Then, each data point is reclassified into five classes by assigning a score. Reclassification is performed using a quantitative classification method [37]. Subsequently, the five parameters are summed to obtain the atmospheric observatory index, as shown in Eq 3. The information in Eq 3, IAO CF, AOD, PWV, WS, and PR, includes the atmospheric observatory index, cloud cover, aerosol optical depth, water vapor, velocity wind, and rainfall, respectively. Spatiotemporal variation is determined by averaging each atmospheric parameter in 2019–2021 monthly. Then, the monthly average is assigned a score based on the results of the previous reclassification. Subsequently, these five parameters are added together to obtain the monthly observatory atmospheric index according to Eq 3. In addition, the results of the atmospheric observatory index can be further analyzed every month to obtain spatiotemporal variation results.

$$IAO=\sum_{i=0}^{m}\sum_{i=0}^{n}{CF}_{i}+{AOD}_{i}+{PWV}_{i}+{WS}_{i}+{PR}_{i}$$ (3)

The observatory suitability index results from the integration of the physical index (IFO) and the atmospheric index (IAO), which can be calculated based on Eq 4, where IKO is the observatory suitability index. The latitude and longitude zones can be determined by integrating the administrative boundary data. These data are used to obtain the longitude and latitude ranges of Indonesia. The division of zones, which are the longitude and latitude zones, is carried out separately. The longitudinal zone is divided into ten equal zones based on the longitude range of Indonesia. The latitude zones are also divided into ten equal zones based on the latitude ranges in Indonesia.

$$IKO=\sum_{i=0}^{n}{IFO}_{i}+{IAO}_{i}$$ (4)

To integrate all data and results from various resolutions, this research uses the min-max normalization method. The min-max normalization method will create a new value in the range of 0–1 by performing a linear transformation [38, 39]. The normalization method used can be seen in Eq (5) [39] with *X*_*t*_ representing the raster value after normalization, *X*_*o*_ representing the value before normalization, *X*_*max*_ being the maximum value before normalization, and *X*_*min*_ being the minimum value before normalization.


$$X_{t}={(X}_{o}-X_{min})/(X_{max}-X_{min})$$ (5)

### Multi-machine learning approach

Another approach in developing the location suitability of observatories is by using a multi-machine learning approach, namely random forest (RF) and gradient tree boost (GTB). The observatory locations in Indonesia are used as training and validation data with input variables consisting of all data in the atmospheric environmental and physical socio-economic aspects group. Random forest is a machine learning method based on response variables [40]. The random forest algorithm consists of a classification model in the form of a decision tree regression and a set of parameters in the form of independent random vectors that are identically distributed with the independent variables x used to create prediction models for each decision tree [41]. Random forest has a mathematical formula used to calculate prediction models, shown in Eq 6 [42].

$$\hat{f}(x)={argmax}_{y}\sum_{J=1}^{J}I({\hat{h}}_{j}(x)=y)$$ (6)

Where $\hat{f}(x)$ represents the probability of random forest, *argmax*_*y*_ is used to determine the class with the greatest predictive probability, *J* represents the sample, *I* represents the number of data, ${\hat{h}}_{j}$ is the predicted class of x, ${\hat{h}}_{j}(x)=y$ is an indicator function, and x is the predictors and y is the response.

The next algorithm, Gradient Tree Boosting (GTB), is a machine learning algorithm that optimizes the classification and regression process based on error functions. The result of gradient tree boosting is a strong prediction model because it integrates low-level prediction models, such as decision trees [43]. The main approach in gradient tree boosting is to reduce the residual result of the last prediction model according to the direction of the gradient of the reduction in residual values [44]. The prediction model used in gradient tree boosting is designed to reduce residual and overfitting with a mathematical formula shown in Eq 7.

$$f_{m}(x)=f_{m-1}(x-1)+lr*\rho_{m}g_{m}(x)$$ (7)

Where, f_m_ represents the probability of GTB, f_m−1_ represents the negative gradient value of the loss function, x represents the predictors, *lr* represents the learning rate, g_m_ represents the loss function, and ρ_m_ represents the regression tree for g_m_.

Furthermore, in creating the suitability index of observatory locations, voting is performed from the two machine learning models to obtain the optimal observatory location using the calculation in Eq 8. The integration of two different machine learning algorithm prediction models, random forest and gradient tree boosting, will result in a new suitability index of the optimal observatory location.

$$IKO_{ML}=\sum_{i=0}^{n}f_{m}(x)+\hat{f}(x)$$ (8)

Where, IKO_ML_ represents the product of the suitability index of observatories based on machine learning, while f_m_ and $\hat{f}$ represent the probability results of the random forest and gradient tree boosting machine learning models, respectively, with *X*, *n*, and *i* representing the number of predicted data, number of data, and the sample representation. Moreover, the suitability index is a probability value derived from IKO_ML_, which is the integration of two machine learning techniques. The index ranges from 0 to 1, where a value of 1 shows a highly suitable location for observatories, while a value of zero indicates an unsuitable location.

## Results

### Physical and socio-economy factor observatory suitability index

**Fig 3:**
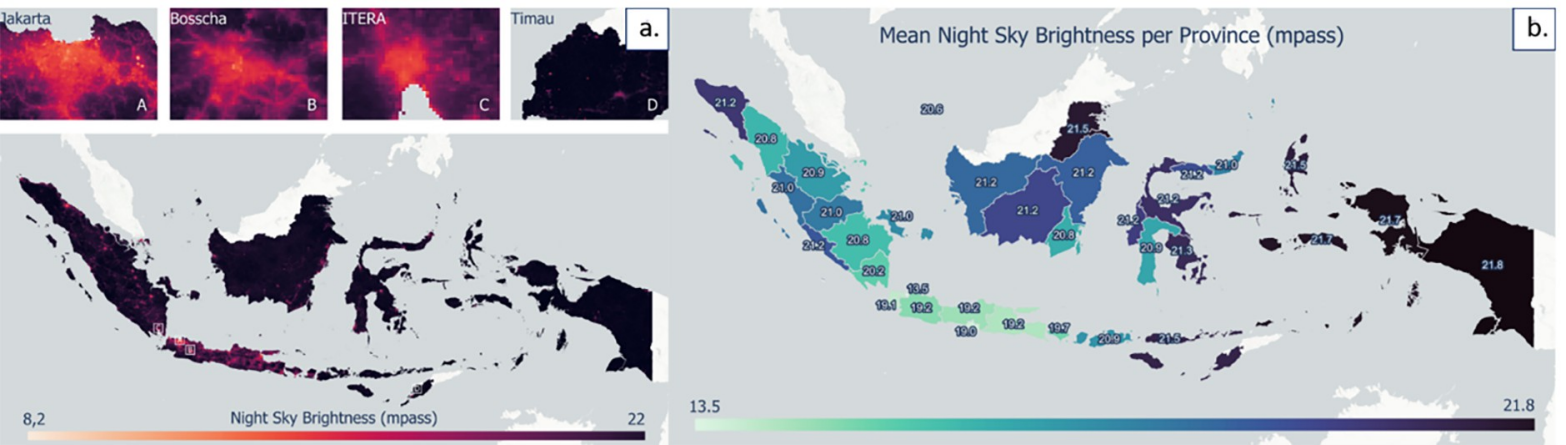
Product of the night sky clarity level: a) Distribution of the night sky clarity in Indonesia and several specific areas such as A. Greater Jakarta, B. Bosscha Observatory, C. ITERA Astronomical Observatory (IAO), D. The National Timau Observatory, and b). Average value of the clarity of the night sky in each province in Indonesia.

**Fig 4:**
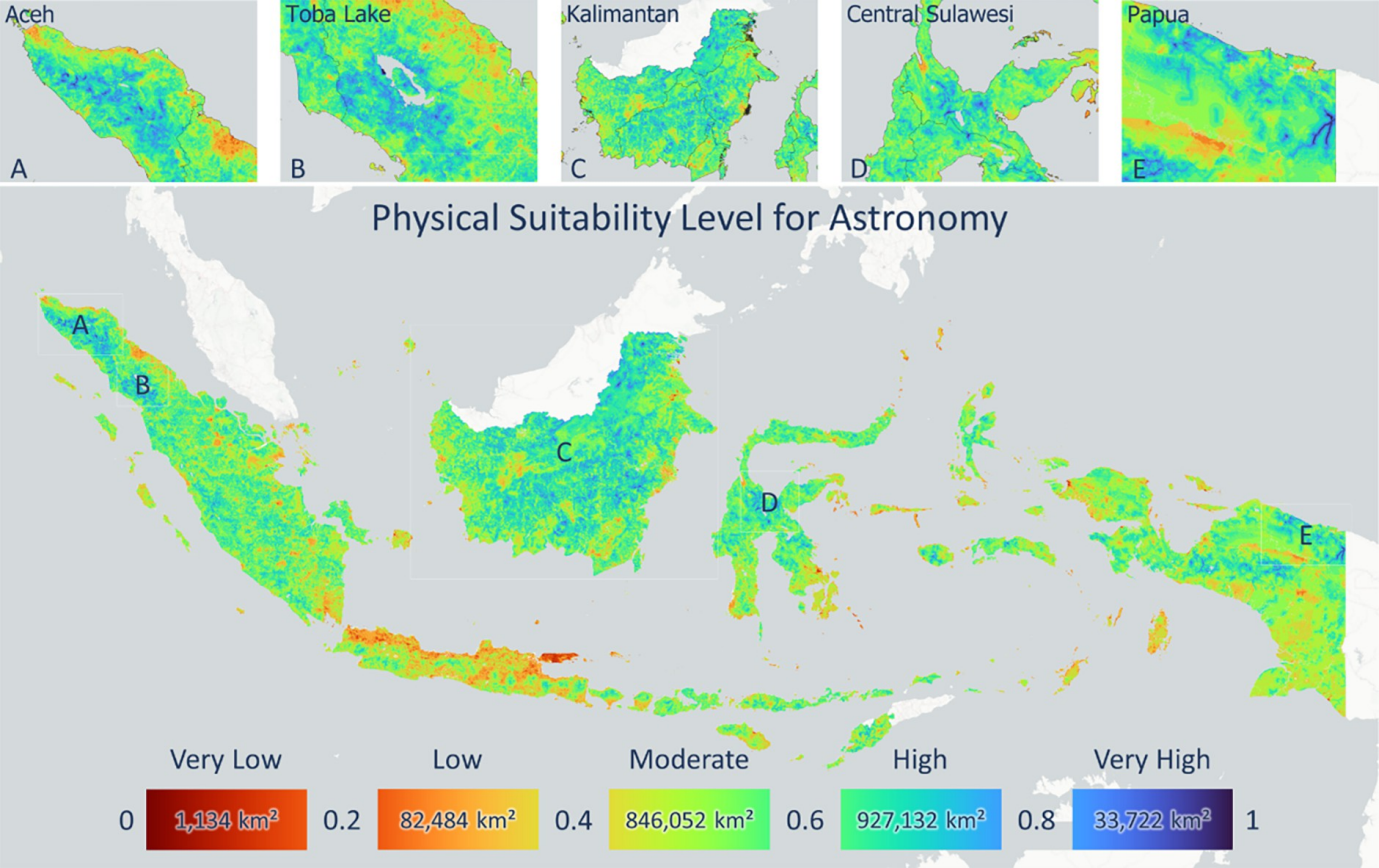
Observatory suitability index based on a combination of the Earth’s physical and demographic–economic parameters in Indonesia, with several provinces with high index values such as A. Aceh, B. North Sumatra, C. East Kalimantan, D. Central Sulawesi, and E. Papua.

The observatory’s physical index indicates a suitable location based on the Earth’s physical, demographic, and economic factors (Fig 3). As an illustration, a high observatory suitability index value based on physical factors is supported by high elevation and high values for road access and electricity but low values for population parameters and light pollution. Fig 3A shows the NSB level product in mpass units, based on calculations from the average VIIRS night light image data for 2021. Areas with low values for NSB indicate that high light pollution can interfere with astronomical observation. Most of Indonesia’s territory remains dominated by high NSB, except for some urban areas, especially in Java and Sumatra, such as Greater Jakarta and Greater Bandung. Fig 3B shows the average NSB score at the provincial level. In general, eastern Indonesia was dominated by areas with NSB values greater than 21 mpass, where Papua Province was the highest province with a value of 21.8 mpass. East Nusa Tenggara Province, Lampung Province, and West Java Province had average values of 21.5 mpass, 20.2 mpass, and 19.2 mpass, respectively, and are the sites of the current National Timau Observatory construction, the IAO, and the Bosscha Observatory. In comparison, Greater Jakarta, ranked the lowest, with an average value of 13.5 mpass. The NSB product was then used to develop a physical index of the total observatory location suitability by integrating it with population data, human activities, road and electricity access, and elevation. Fig 4 shows the product of the observatory location suitability index based on physical factors. Overall, Indonesia is dominated by moderate and high suitability classes with an area of roughly 1.75 million km^2^. Locations with a very high index are shown in blue with an area of 33,700 km^2^; conversely, locations with a very low index are shown in red. The island of Java is dominated by very low and low class owing to its high population and human activity. In addition, the location of big cities on Java also causes the night sky to be contaminated by light pollution, so it is not suitable for the location of the observatory.

### Atmospheric environmental factor observatory suitability index

**Fig 5:**
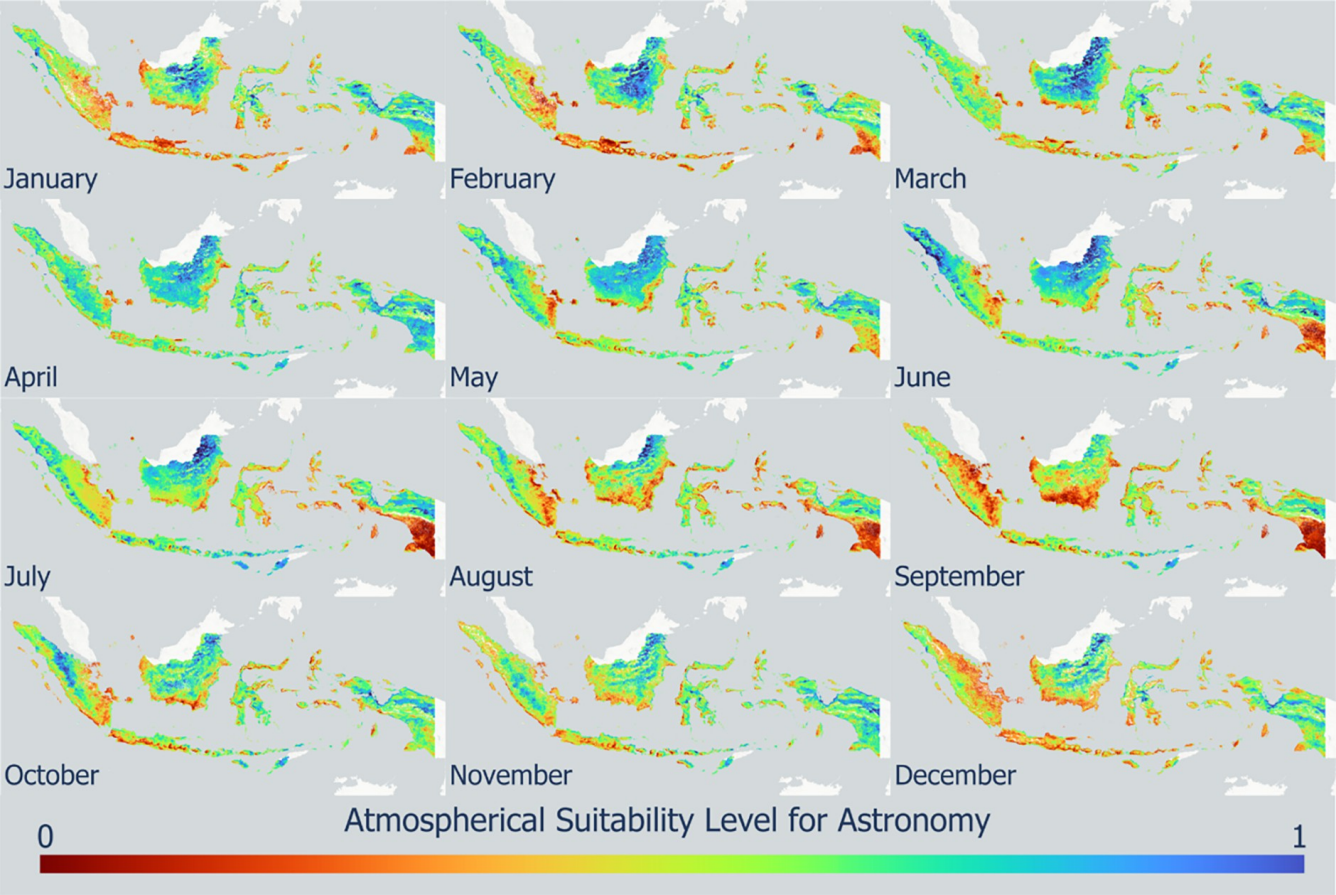
Spatio-temporal variation in the average monthly atmospheric index value distribution in 2019–2021.

**Fig 6:**
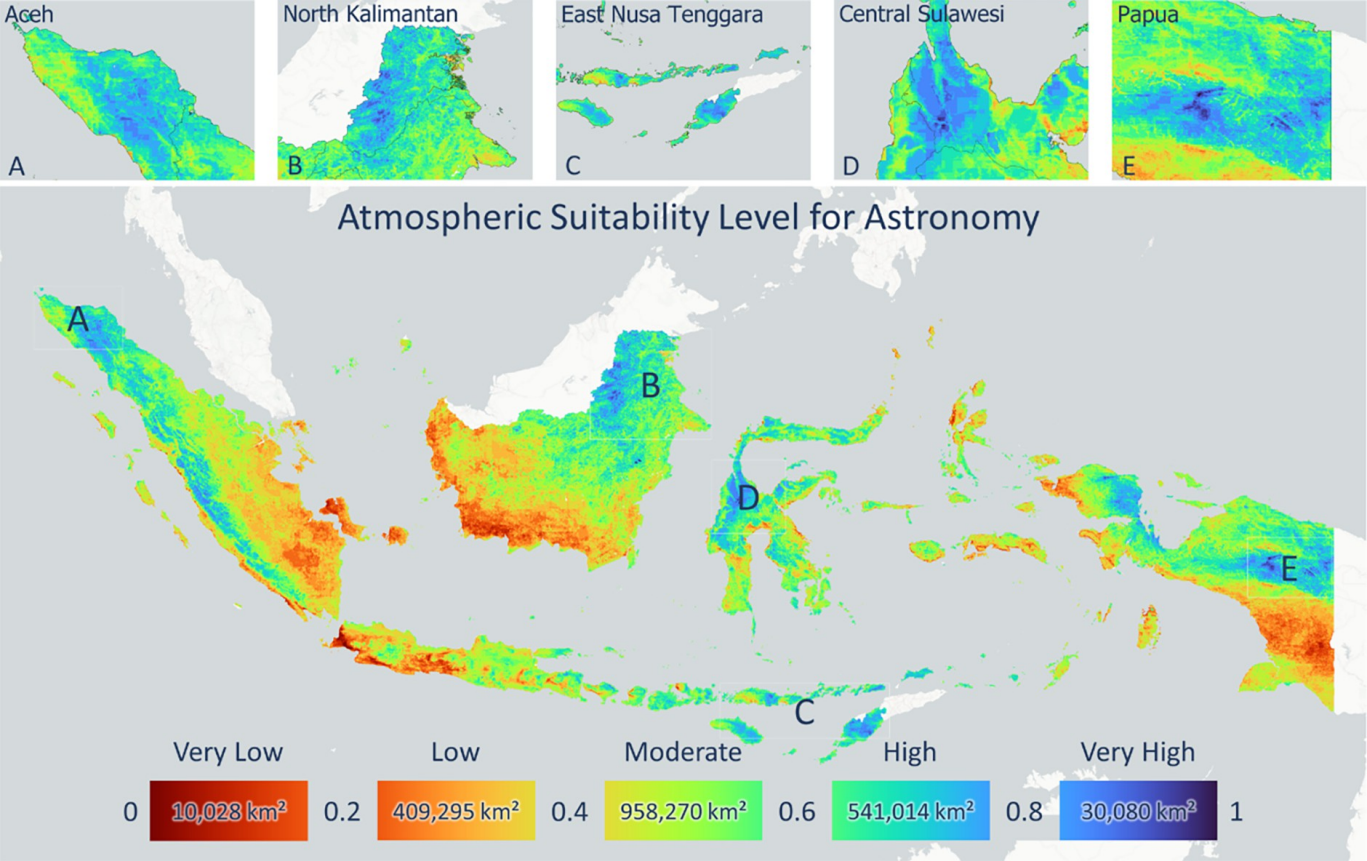
Total atmospheric factor index, with several provinces with high index values, such as A. Aceh, B. North Kalimantan, C. East Nusa Tenggara, D. Central Sulawesi, and E. Papua.

The atmospheric index product in this study was developed based on cloud cover, AOD, PWV, wind speed, and rainfall data. Fig 5 shows the spatiotemporal variation in the atmospheric index from the 2019–2021 monthly mean values. Indonesia is generally dominated by a fairly high level of conformity between April and May. The central to northern Kalimantan islands had a very high level of conformity from January to August, as shown in blue. The northern part of Sumatra Island had a fairly high level of suitability from May to June. The central and northern parts of Papua Island showed a fairly high level of conformity from January to July and continued from October to November. The southern part of Papua Island had the lowest suitability level from June to September. A low level of conformity dominated the southern parts of Sumatra and Java, from December to January. Timor Island, East Nusa Tenggara (NTT), showed a very high level of conformity from April to November. Fig 6 shows the total atmospheric factor suitability index. Provinces with a high atmospheric index included Aceh, North Kalimantan, East Nusa Tenggara, Central Sulawesi, and Central Papua with a total area of 30,000 km^2^. Southern Papua, the west to the south side of Kalimantan Island, the west side of Java Island, Central Java, and the southeast side of Sumatra Island had low total atmospheric index values with a total area of 420,000 km^2^, so they are not suitable for the construction of astronomical observatories. Several conditions, such as poor air quality due to trends in forest fire activity, and anthropogenic sources that increased the AOD value and high cloud and rain intensity in several areas, caused a low value of the observatory suitability index for this atmospheric factor.

### Multi-machine learning-based observatory suitability index

**Fig 7:**
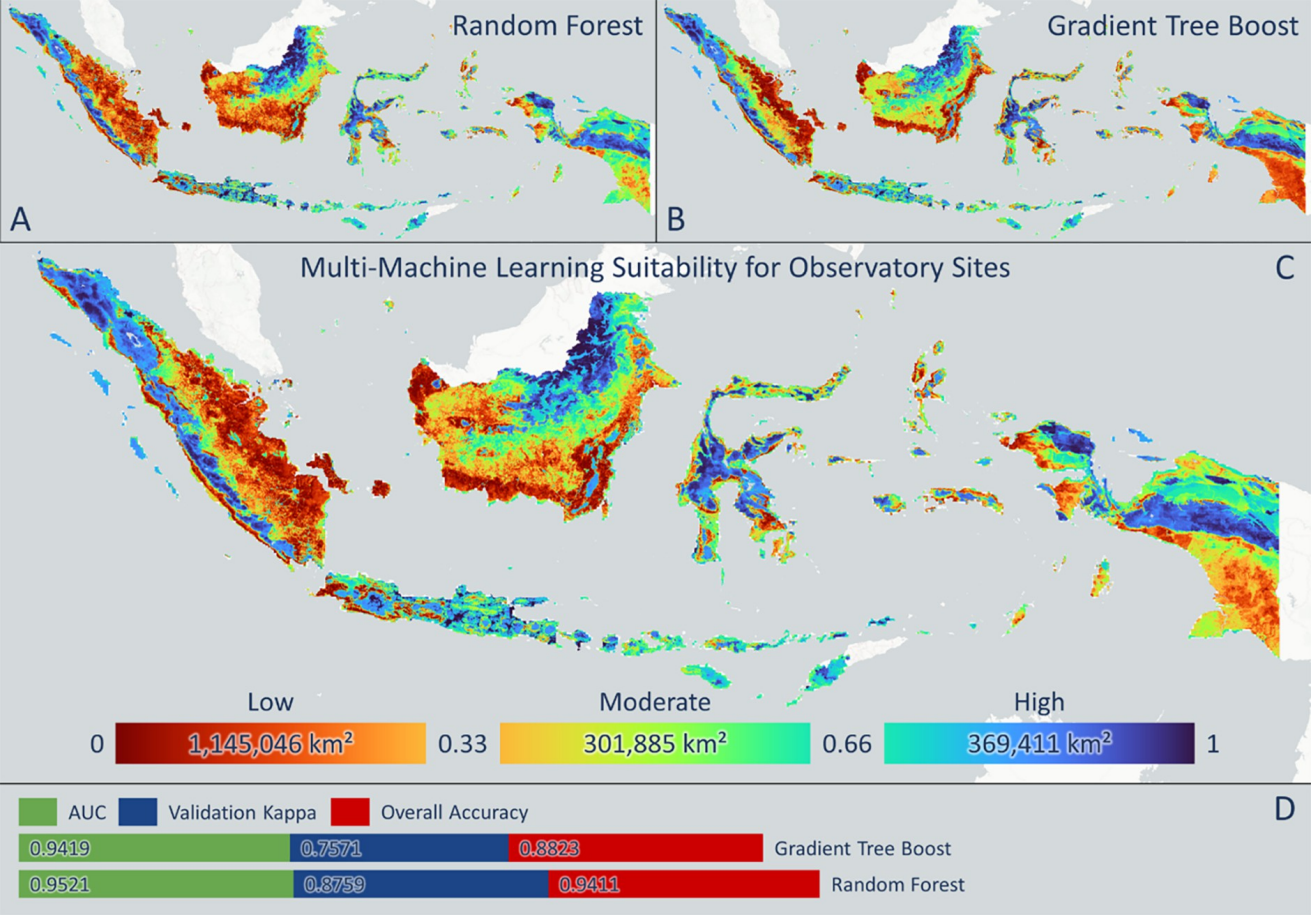
The results of a machine learning-based analysis of suitability levels for constructing observatories: A. displays the results of the random forest (RF) algorithm, B. presents the outcomes of the Gradient Tree Boost (GTB) algorithm, C. illustrates the combined product of both machine learning approaches, and D. shows the statistical results including AUC, validation kappa, and overall accuracy.

Fig 7 presents the outcomes of machine learning techniques combining random forest and gradient tree boosting. The highest probability for potential observatory sites is distributed throughout the mountainous regions of Indonesia with an area of roughly 369,000 km^2^. In contrast, the least suitable areas for observatories are primarily found along the coastlines of islands such as Kalimantan and Sumatera. This is likely due to the low elevation of coastal areas, which makes them unsuitable for observatories. Based on the results of the random forest algorithm, the top five provinces with the highest potential suitability for observatories are Yogyakarta, Bali, East Java, Aceh, and Central Sulawesi, respectively. Meanwhile, according to the gradient tree boost algorithm, the top five regions with the highest potential suitability are Aceh, Bali, North Sumatra, Yogyakarta, and Central Sulawesi, respectively. Overall, both approaches recommend the same five provinces. However, there are differences in the results for two provinces, namely North Sumatra and East Java, which rank differently in the two machine learning algorithms. East Java ranks third highest in the random forest algorithm but seventh highest in the gradient tree boost algorithm, while North Sumatra ranks third in the random forest algorithm and 18th in the gradient tree boost algorithm. These discrepancies may be influenced by the different algorithms used in each approach. The agreement between the two machine learning algorithms suggests that, in order of suitability, the regions of Aceh, Bali, Yogyakarta, East Java, and Central Sulawesi are highly recommended as potential observatory sites for astronomy. Furthermore, the accuracy of the algorithms is measured using Overall Accuracy, Validation Kappa, and Area Under Curve (AUC). The Random Forest algorithm demonstrates good accuracy for identifying suitable areas for observatories, with percentage values of 0.9411 for Overall Accuracy, 0.8759 for Validation Kappa, and 0.9521 for AUC, respectively. Conversely, Gradient Tree Boost performs with lower accuracy than Random Forest, as indicated by the following values: 0.8823 for Overall Accuracy, 0.7571 for Validation Kappa, and 0.9419 for AUC.

### Distribution of optimal observatory construction

**Fig 8:**
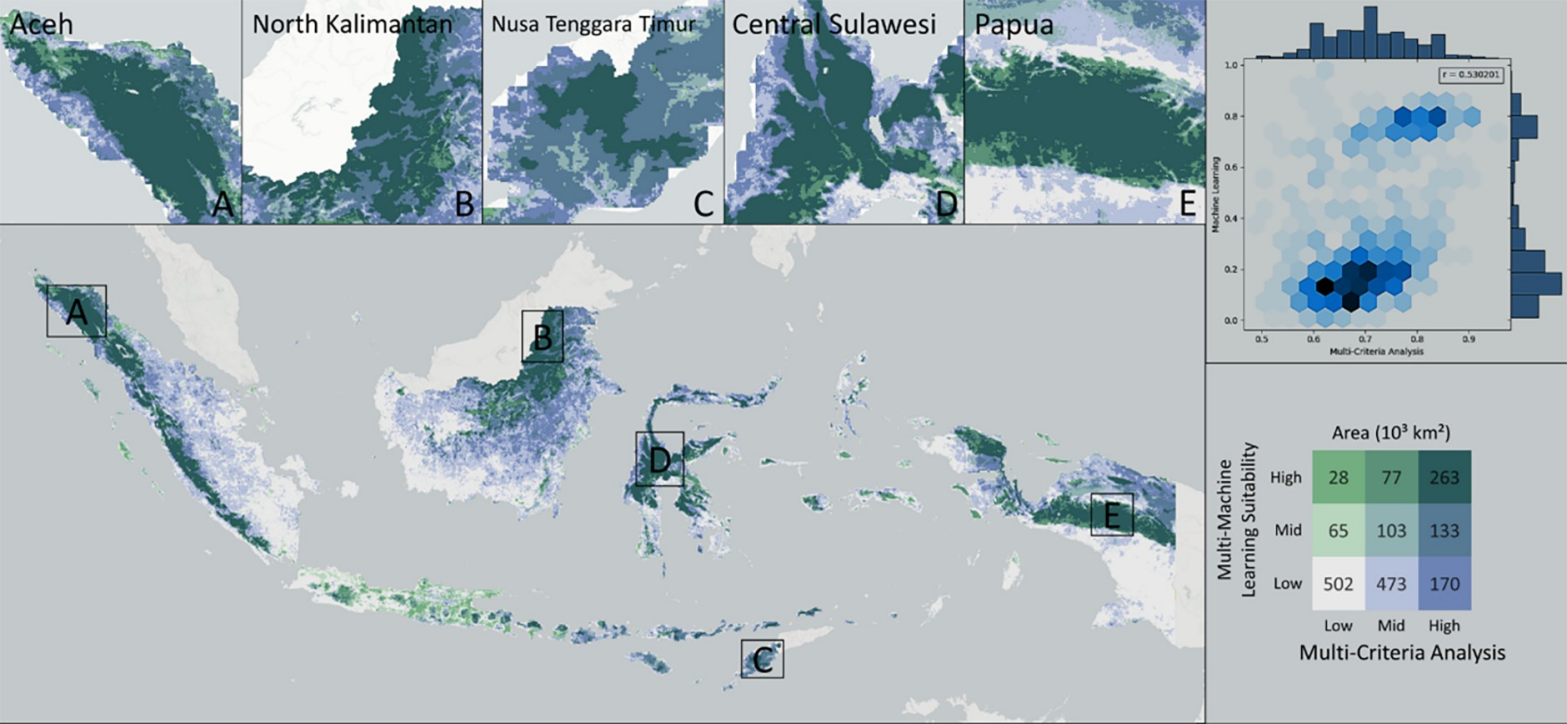
The analysis of agreement between two different approaches to assess the suitability of observatory sites: Multi-criteria and the multi-machine learning approach.

The map shows the bivariate result between the multi-machine learning and the multi-criteria analysis results (Fig 8). The light grey color, which accounts for the largest area (over 500 thousand square km), illustrates the low MML and low MCA, followed by the light blue color, which makes up for 473 thousand square km, demonstrating the low MML and mid-MCA. While the dark green color, which illustrates the highest suitability for observatories in both methods, accounts for the third largest area just above 260 thousand square km. Based on the maps, locations with the highest potential are located on the mountain ranges across Indonesia (Sumatera, Kalimantan, Sulawesi, and Papua) and a few are located on the dry climate location of Indonesia (Nusa Tenggara). Based on the given graph, it is shown that the correlation value (r-value) between the ML method and the MCA method is 0.53, which illustrates a moderate-strong correlation. It can be clearly seen that the data are densely distributed between the low values (0–0.2) of the ML method and the moderate-high values (0.6–0.7) of the MCA method, which is illustrated by the dark blue color and moderately spread between the high values of both methods, which demonstrated by the blue color.

**Fig 9:**
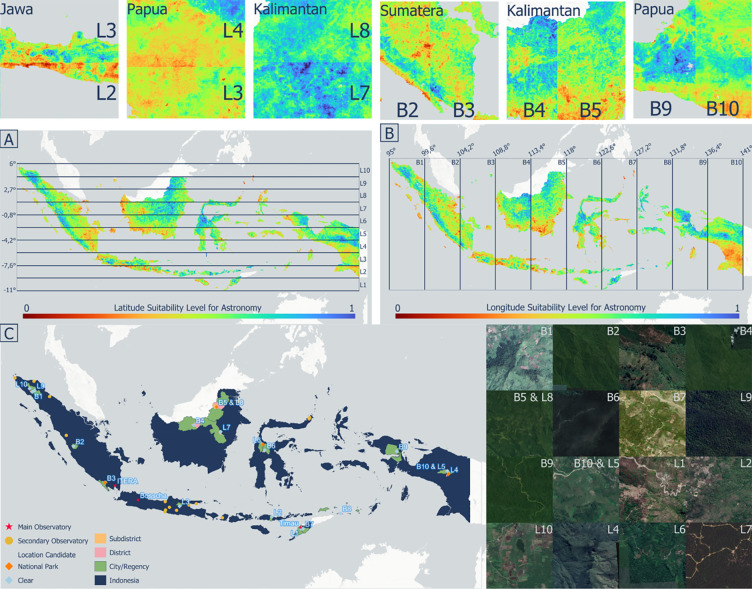
Combined observatory suitability index distribution zoning based on A). Latitude Zone (L1–L10), B). Longitude Zone (B1–B10) and C. Optimal distribution of observatory scenario: Locations of eighteen recommendations for the construction of a new Indonesian observatory.

The product of the combined observatory suitability index was the result of integrating the results of the previously developed observed multi-criteria analysis (physical, atmospheric, environmental) and multi-machine learning (Fig 9). To determine the distribution more optimally and evenly throughout Indonesia, ten zones were divided based on longitude and latitude. Thus, each zone had an area with the highest and lowest relative conformity index values. Generally, the high-index provinces were Aceh, North Kalimantan, East Nusa Tenggara, Central Sulawesi, and Central Papua. In contrast, the provinces with a low index were dominated by the provinces of Java Island, southern Papua, the west and south coasts of Kalimantan Island, and the coastal areas east of Sumatra Island. Fig 9A and 9B show the combined observatory suitability index of physical and atmospheric factors divided by the ten latitude zones (L1–L10) and ten longitude zones (B1–B10), respectively. The division of zones shows a clear color difference on Java Island at the L2–L3 line boundary, Papua Island at the L3–L4 line boundary, and Kalimantan Island at the L7–L8 line boundary (Fig 9A). The zone division also shows a clear difference in values on Sumatra Island at the B2–B3 line boundary, Kalimantan Island at the B4–B5 line, and Papua Island at the B9–B10 line boundary (Fig 9B). This proves that the zone division shows significant results in determining the appropriate location relative to the longitude and latitude zones that have been formed.

A total of eighteen locations were recommended for building astronomical observatory stations. The recommended location naming uses latitude–longitude zone division information, B1–B10 for naming locations based on longitude zones, and L1–L10 for naming locations based on latitude zones (Fig 9C). Two locations were created by the intersection of the two zones, B5–L8 and B10–L5. In addition, four locations were located in national parks: B3, B5–L8, L4, and L6. There were five recommended locations on the island of Sumatra (L10, L9, B1, B2, and B3), one on Java (L3), three on the island of Kalimantan (B5–L8, B4, and L7), four on Nusa Tenggara (L2, L1, B7, and B8), two in Sulawesi (L6, B6), and three in Papua (B9, B10–L5, L4). Of the three main observatory locations in Indonesia, namely Bossca, ITERA, and Timau (marked by an asterisk), only Timau is included in the prioritized point category in Bandung, in the L1 zone. Information about the administrative area from the recommendation point, namely district, sub-district, and city/regency, is displayed to prepare for future budget administration and planned infrastructure.

## Discussion

### Machine learning statistic evaluation

**Fig 10:**
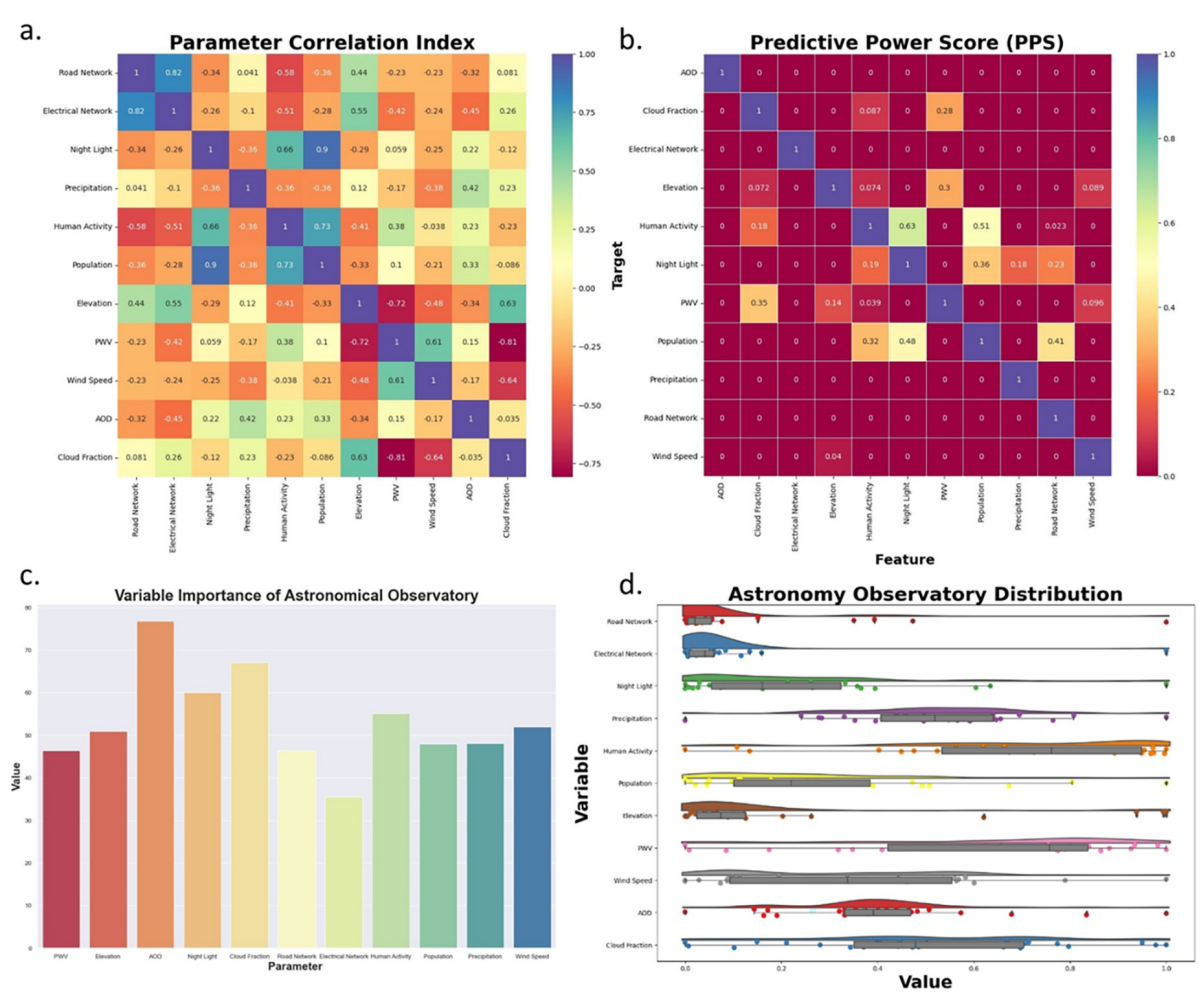
Machine learning statistical evaluation: a. Parameter correlation matrix, b. Predictive power score, c. Variable importance of astronomical observatory and d. distribution value of the existing observatory site to each parameter.

To analyze the results of the machine learning product, several analyses were performed, including the parameter correlation matrix, predictive power score (PPS), variable importance, and the distribution value of existing observatory sites to each parameter (Fig 10). The correlation matrix analysis was used to identify the relationship between variables using the Pearson correlation test, which produces correlation values ranging from -1 to 1, indicating that values closer to 1 or -1 have a high degree of correlation. Fig 10A shows the distribution of values dominated by low to moderate groups, where 72.7% of the correlation values indicate low to moderate values. This suggests that the data used in this study can be used as input parameters without any data needing to be eliminated. The correlation value between population and night lights had the highest correlation value of 0.9, indicating that the population in an area can be identified through the brightness of the night lights. This is because the brightness of the night lights can identify socioeconomic activity. The higher the value of the night lights, the higher the level of socioeconomic activity in that area [45, 46]. Therefore, with a correlation index parameter value dominated by low to moderate values, the variables used are already good.

In this study, a PPS test was also performed, as shown in Fig 10B, which indicates the level of independence between the variables used [47]. The PPS analysis was used to support the correlation index parameter analysis, where a value of 0 indicates that variable x cannot predict variable y. In addition, values close to 1 indicate a strong relationship for predicting variable y. The variable that had the highest Predictive Power Score value was the night lights, which was very strong in predicting human activity with a score of 0.64. However, the reverse was not true, indicating that human activity had a small impact on predicting night light values, with a score of only 0.19. In addition, the road network variable could predict population values well with a score of 0.41. However, the population had no influence on predicting the value of the road network in an area with a PPS value of 0.

After analyzing the relationship between input variables using the correlation matrix and PPS approaches, the next analysis that can be performed is to calculate the variable importance among the 11 input parameters in determining the suitability level of observatory locations (Fig 10C). AOD, night lights, and cloud fraction were found to be the three parameters with the highest important values in the data analysis process. On the other hand, electrical and road network parameters were the two inputs with the lowest important values. These important values were influenced by the distribution of training data points on input variables, as shown in Fig 10D, which directly affects the suitability of observatory products created through both machine learning algorithms. The diverse distribution of values from the training points on the input variables indicates that the quality of the existing astronomical observatory distribution generally has various conditions. This can be understood that the placement of observatory locations, especially in Indonesia, is generally influenced by the assets owned by observatory owners. In this case, the placement of observatory locations has not always considered various other considerations, including physical aspects of the earth, environmental and meteorological conditions.

### Future observatory preparedness using multi-risk air pollution

In carrying out the distribution and recommendations for optimal observatory development, apart from having a high composite index of suitability, another consideration is the low risk of air pollution. The risk of air pollution, whether caused by human factors such as forest fires, transportation, power generation, and mining activities, or natural factors such as volcanic eruptions and fires due to drought, is a phenomenon that needs to be avoided in determining the location of future observatory developments. In this study, the multi-risk product of air pollution developed in previous studies was used [34] to determine potential observatory locations that are free from the risk of air pollution disturbances.

**Fig 11:**
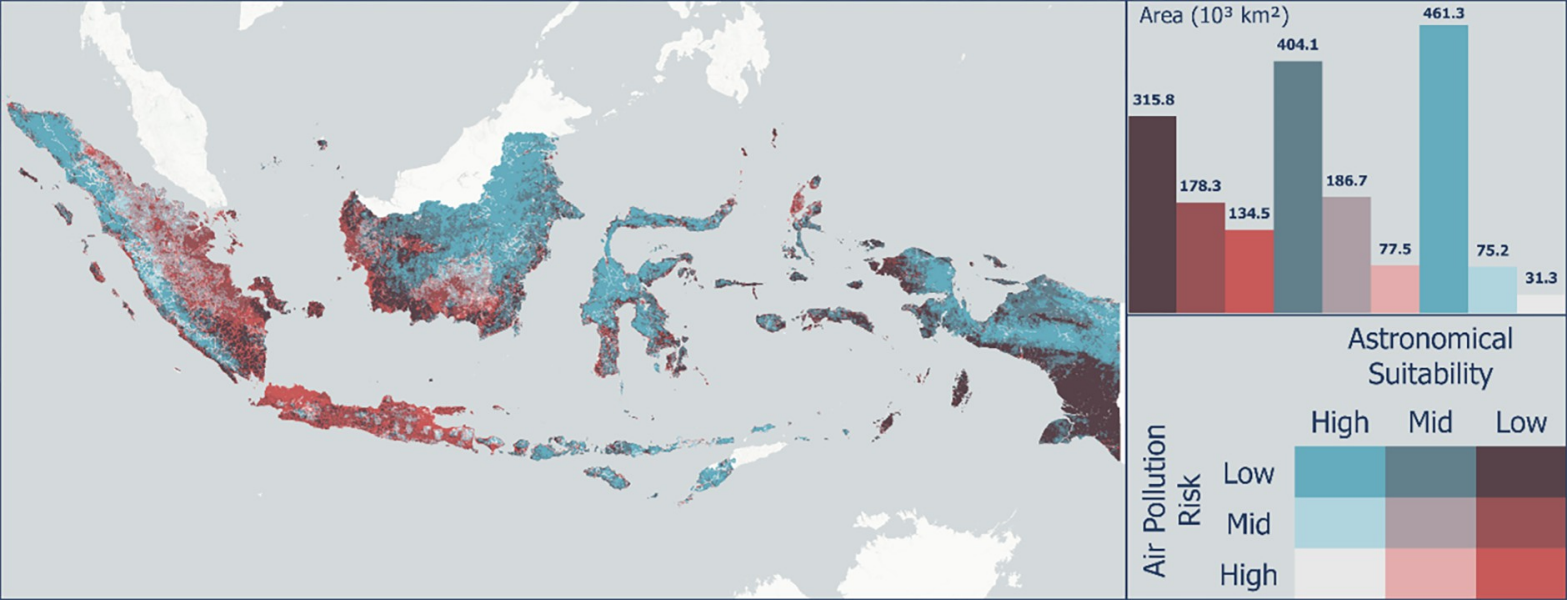
Bivariate visualization between astronomical observatory suitability index and multi-air pollution risk product.

Fig 11 shows the integration of the combined observatory suitability index results with multiple risk indices of air pollution. In general, Indonesia is dominated by areas with minimal air pollution, so the potential for determining favorable observatory locations remains quite high, and the widest area is indicated by the "high astronomical suitability-low air pollution risk" class with an area of 461.3 thousand km^2^. The identified condition is found in several areas, including the mountainous area of Sumatra, northern Kalimantan, Central Sulawesi, Timor Island, and northern Papua Island. This condition must be maintained by continuously controlling air pollution levels so that the potential locations for observatory activities do not decrease. Most of Java Island, the east coast of Sumatra Island, and the west and south coasts of the island of Kalimantan are included in the "low astronomical suitability–high air pollution risk" class, covering an area of 134.5 thousand km^2^. Java is a densely populated island, thus numerous anthropogenic activities could potentially be emission sources e.g., motor vehicles, industries, and commercial and residential activities. The contribution of those emission sources in Jakarta, the capital and largest city of Indonesia located in Java, has been studied by Lestari et al. [48]. Meanwhile, forest fire has frequently occurred in Kalimantan and Sumatra because of land clearing for agriculture and settlements. It has also been considered as a significant emission source that contributes to the air quality in those areas. There is also an area of 106.5 km^2^ that is included in the high astronomical suitability category but is in the high-medium air pollution risk class, which is not recommended in the priority areas for the development of Indonesian observatories.

### Main observatory characteristic

**Fig 12:**
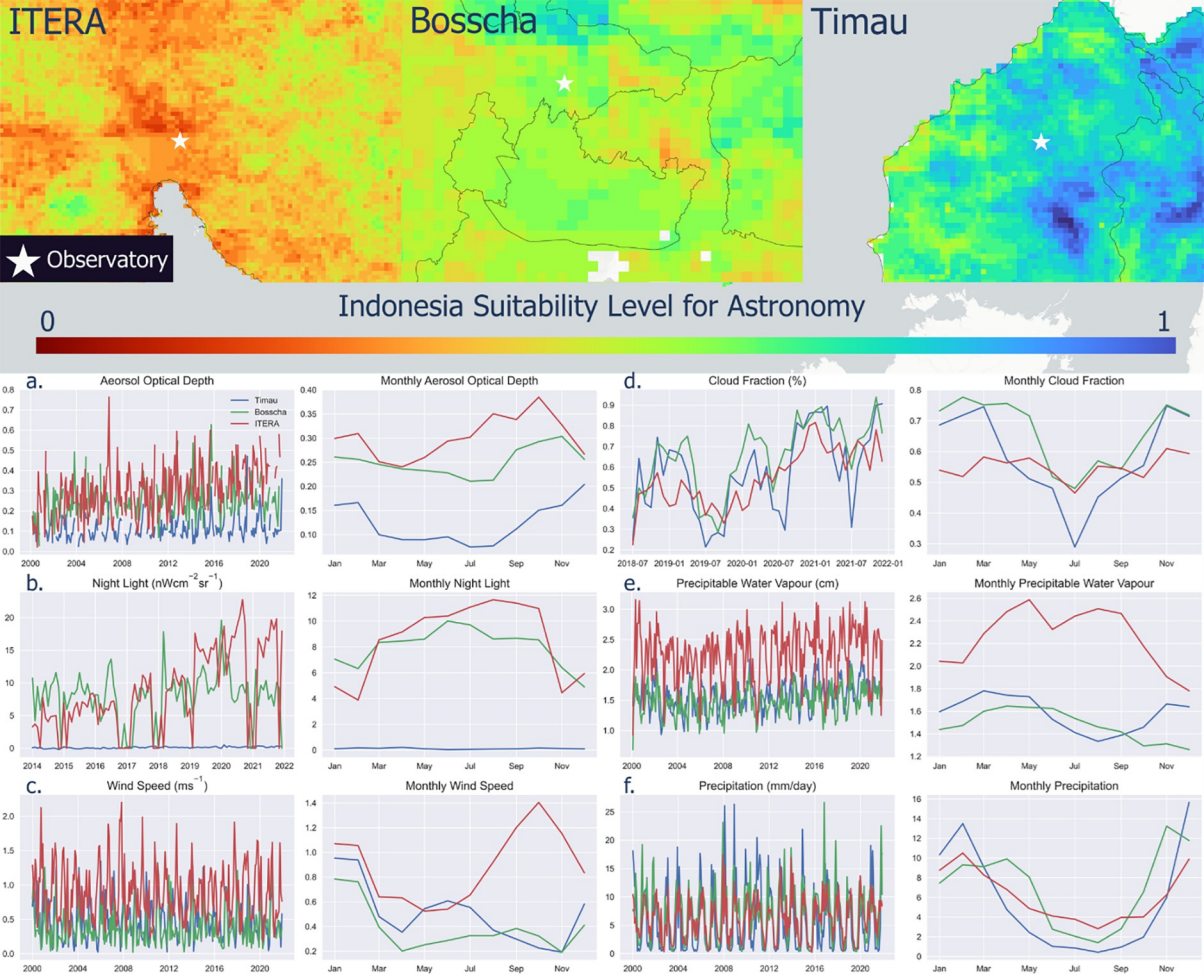
Index comparison results for three main observatory locations: a). Aerosol optical depth, b). Night light, c). Wind speed, d). Cloud cover, e). Precipitable water vapor, and f). Rainfall.

Long-term investigations of the observatory conformity index product at three main observatory points, Timau, Bosscha, and ITERA, are shown in Fig 12. The conformity index product was developed based on the accumulation of combinations of various long-term data parameters. The area of the Timau Observatory is shown to have a high suitability value, which agrees with Hidayat et al. [3], who stated that Timau is a location with a high degree of suitability for astronomical observatory activities. The Bosscha Observatory area is moderately suitable due to light pollution related to massive economic and infrastructure development; therefore, the clarity of the night sky has decreased [10]. The IOA area is dominated by low color conformity, which is caused by its location in an urban area. Therefore, it is affected by direct light pollution. This is exacerbated by the poor air clarity condition caused by air pollution aerosols. To understand the dynamic pattern of significant changes in various parameters at the three main observatories, a long-term time series analysis was conducted on six parameters: AOD, night light, wind speed, cloud fraction, PWV, and precipitation. Time series visualization in daily and monthly units for these six parameters is shown in Fig 12A–12F, where the blue lines show The National Timau Observatory, the Bosscha Observatory is in green, and ITERA is in red. Parameters such as AOD and night light are influenced by human activities. This situation can be suppressed by taking precautions such as regulating the use of night lights, as well as reducing air pollution caused by human activities such as vehicle emissions, industries, and forest fires. However, parameters such as wind speed, cloud fraction, PWV, and precipitation are natural parameters whose conditions are difficult or irreversible and depend on time. Therefore, astronomical observations must be adjusted to the time and the most optimal natural conditions.

From the results of long-term observations, the AOD condition at the National Timau Observatory had a much lower value than at the other two observatories and was relatively stable year-round. Not only the AOD parameter, but the PWV parameter in Timau also had a relatively stable value throughout the year. In addition, the very low and stable night light throughout the year means that the National Timau Observatory is not affected by light pollution; therefore, the sky is dark and optimal for observatory observations. The Bosscha Observatory has the advantage of relatively lower wind speed values than other observatories and tends to be stable from April to August. The cloud cover parameter was relatively higher than that of other observatories and experienced its lowest point from June to July. The rainfall at the Bosscha Observatory has a stable and low value from June to September. PWV in Bosscha is relatively stable throughout the year; therefore, the transparency of the atmosphere is relatively constant. The Timau Observatory has notable advantages of stable and low rainfall occurring from May to October. In addition, the IOA has stable cloud cover, which allows year-round observations. The night light at the IOA shows dark skies in February and November and stable and relatively low rainfall from May to October. However, the IOA had a higher PWV parameter than the other observatories. In addition to PWV, the AOD parameter also tended to be higher than that of other observatories, which could be caused by forest fires in Sumatra. These optimum observation periods could be utilized to organize various astronomical tourism activities and agenda for the corresponding observatories. Therefore, each observatory could promote distinctive observation activities to be offered to the visitors to obtain the best visual experiences.

### Limitations and future study direction

Continued remote sensing technology development in the future will be the key to expanding this study because the data used have a resolution of more than 5 km for rainfall and 11 km for wind speed. Furthermore, there were fewer actual road network and human activity data in 2018 and 2016. In addition, index development could benefit from a longer period, so that the results obtained will be better because this study only used temporal data from 2016 to 2019. The data for 2021 are currently in the form of an atmospheric index. Further studies can validate the resulting index by using DIMM tools and SQM photometers [14]. The use of weights for each parameter can also be employed in subsequent studies, which can be determined based on the priority of each parameter using AHP [18, 49]. Future studies could also consider the optimum and variety of visual experienced (i.e. objects and visibility) obtained from the observations as the criteria in AHP. It will be beneficial to determine the positioning of each observatory in the development of astronomical tourism. Another approach could be to use automated algorithms, such as machine learning for index generation [50]. In addition, other parameters, such as disaster parameters, are also involved in constructing an index to generate recommendations for locations that are not prone to disasters [20].

## Conclusions

This study aimed to develop a scenario for the distribution of optimal locations for the construction of infrastructure for astronomical research observatories in Indonesia. To achieve this goal, several supporting objectives include developing an observatory suitability index based on Earth’s physical, demographic–economic, and atmospheric-environmental factors, identifying the location of observatory construction based on the division of longitude and latitude zones and considering the risk of air pollution disturbances. Java Island is dominated by low physical index owing to its high population and human activity causes the night sky to be contaminated by light pollution. In addition, Southern Papua, the west to the south side of Kalimantan Island, the west side of Java Island, Central Java, and the southeast side of Sumatra Island had low total atmospheric index values due to trends in forest fire activity, and anthropogenic sources that increased the AOD value and high cloud and rain intensity in several areas. Indonesia is dominated by areas with minimal air pollution, so the potential for determining favorable observatory locations remains quite high, and the widest area is indicated by the "high astronomical suitability-low air pollution risk" class with an area of 461.3 thousand km^2^. A total of eighteen locations were recommended for building astronomical observatory stations. Through this study, regulators and astronomers can globally apply a comprehensive approach to determine the best observatory construction location to maximize the research, education, and astro-tourism potential. Furthermore, each of the three existing observatories could promote distinctive observation activities to be offered to the visitors to obtain the best visual experiences. The optimum observation period could be utilized to organize astronomical tourism activity and agenda for these observatories.
